# Finding pediatric thromboembolism: needles in a big data haystack

**DOI:** 10.1038/s41390-024-03186-4

**Published:** 2024-04-20

**Authors:** Katrina Blankenhorn, William Beau Mitchell

**Affiliations:** Albert Einstein College of Medicine, Bronx, NY USA

Thromboembolism is rare in healthy pediatric patients, but it is an increasing problem in children with underlying medical conditions such as cancer. The increase in childhood thromboembolism over the past decade is thought to be due to both heightened awareness of the diagnosis and more invasive technologies used in children with underlying medical conditions.^1,2^ Understanding thromboembolism and developing safe treatment options for pediatric oncology patients is important due to the increased risk of death, organ dysfunction, and poor oncologic outcome. Consequences of thromboembolism also include increased hospital length of stay and cost.^3^ Current published evidence on treatment for thromboembolism in the pediatric oncology population is limited, and published guidelines are often extrapolated from adult trials.^4,5^ For example, in the 2018 guidelines released from the American Society of Hematology for treatment of thromboembolism in the pediatric population, although recommendations were made by a panel of experts, all the recommendations were limited by low or very low certainty in the evidence.^6^

A key step toward gathering better evidence regarding pediatric thromboembolism in the pediatric oncology population will be the development of validated methods for accurately quantifying thromboembolism diagnosis and outcomes. Much current epidemiological research uses administrative data, or “big data,” to identify cases of interest; however, validity of research findings based on these data depend on the validity of the search parameters. Validity depends on both the proper diagnostic coding by physicians and on the proper choice of search codes by the researchers. Current research in the field has been met with various challenges. For example, recent studies to ascertain the rates of childhood thrombosis have relied on discharge diagnosis codes for identifying thromboembolism cases.^2^ However, Burles et al. identified pitfalls of using discharge diagnosis code searches, highlighting the extensive presence of false positives and negatives in identifying thromboembolism cases.^7^ This highlights the fact that healthcare databases, designed primarily for administrative and billing purposes, often lack comprehensive clinical information crucial for research. This includes lack of details on diseases of interest, health outcomes, medications, data on comorbidities, and quality of life.^8^

Addressing the potential of administrative health care databases as validated sources for data, Doiron et al. discussed the benefits of linking large cohort studies with administrative data to enrich datasets, maximize resource utilization, and facilitate multidisciplinary research.^9^ Additionally, regular validation studies, evaluating different code combinations or algorithms, are crucial for ensuring data accuracy, particularly in pediatric populations where such studies are limited.^10^ In the current manuscript, Athale et al. tested the validity of using combinations of ICD and medication codes from large Canadian administrative databases, with a curated oncology database for case verification, to identify thromboembolism diagnoses in children undergoing primary cancer therapy. Multiple query algorithms were tested and validated using the oncology database. The best performing algorithm resulted in a sensitivity of 76% and specificity of 86% for identifying pediatric oncology patients with thromboembolism. Of note, the same analysis improved sensitivity to 84% when using exclusively ICD-10 codes, highlighting the previously reported limitations in using ICD-9 codes for epidemiological research^11^.

This study demonstrates the validation of search parameters for accurately identifying thromboembolism cases in pediatric populations undergoing cancer therapy using multiple administrative databases in conjunction with a large oncology database. These findings could be instrumental for future epidemiological and outcomes research in this area. Future research will be needed to validate this algorithm in other health care systems. Further validation research can also extend this algorithm to other populations such as neonates, or those with other high-risk conditions for thromboembolism. Such studies would test the algorithm’s generalizability and applicability in diverse clinical settings. Outside of the thromboembolism field this study can serve as a model for validation strategies for big data research in other diseases.
